# Eco-Friendly Synthesis of Cerium Nanoparticles Using Spirulina platensis: Assessing Antibacterial and Anti-inflammatory Efficacy

**DOI:** 10.7759/cureus.71502

**Published:** 2024-10-14

**Authors:** Mathesh A, Ramanathan Snega, P Geetha Sravanthy, Muthupandian Saravanan

**Affiliations:** 1 Department of Pharmacology, Antimicrobial Resistance (AMR) and Nanotherapeutics Lab, Saveetha Dental College and Hospitals, Saveetha Institute of Medical and Technical Sciences (SIMATS), Chennai, IND

**Keywords:** antibacterial activity, anti-inflammatory, ceo-nps, environmental friendly, green synthesis, spirulina

## Abstract

Introduction: *Spirulina platensis, *a type of cyanobacterium (blue-green algae), is well known for its rich abundant nutritional profile and bioactive compounds, which contribute to various biological functions within the human body. The application of nanotechnology to Spirulina has the potential to further enhance its biological activity in biomedical assays.

Objective: This study aimed to utilize *Spirulina platensis *for the green synthesis of cerium oxide nanoparticles (CeO-NPs) and evaluate their physiochemical properties. The research will assess the antibacterial and anti-inflammatory efficacy of the synthesized nanoparticles and explore the underlying mechanisms of action.

Methodology: *Spirulina platensis*-mediated* *cerium oxide nanoparticles* *are synthesized by the green synthesis (titration method). The biosynthesized CeO-NPs are characterized by using techniques such as UV-visible spectroscopy (UV-Vis), Fourier transform infrared spectroscopy, X-ray diffraction spectroscopy, scanning electron microscopy (SEM), and energy-dispersive X-ray (EDX). Antibacterial activity was carried out by the agar-well diffusion method and anti-inflammatory activity was carried out by the albumin denaturation method.

Result: The green synthesis of cerium oxide nanoparticles (CeO-NPs) using Spirulina, a sustainable and eco-friendly method has potential application in antibacterial and anti-inflammatory therapies. This study focuses on the green synthesis of CeO-NPs and characterizes them by using UV-Vis, Fourier transform infrared spectroscopy (FT-IR), X-ray diffraction analysis (XRD), SEM, and EDX. The UV-vis analysis confirmed the presence of CeO-NPs at a wavelength of 320 nm. FT-IR reveals four functional groups, such as C-O, N-O, and C=C stretches. XRD analysis showed higher crystalline and less amorphous content. SEM and EDX spectra were utilized to confirm the morphology (agglomerated square shape) and the elemental composition [Ce, O, C] in the CeO-NPs. The antibacterial activity was evaluated against multidrug-resistant (MDR) clinical strains and the anti-inflammatory activity revealed significant activity in a dose-dependent manner.

Conclusion: This study concluded that Spirulina-mediated CeO-NPs have potential as a drug in biomedical assays. Further in vitro and in vivo analysis is required to fully confirm their viability as a potential drug.

## Introduction

Nanotechnology is a fast-growing, multidisciplinary field with many applications in science and technology [1]. The development of new techniques for handling and synthesizing nanoparticles (NPs) involves combining ideas from various fields such as chemistry, engineering, physics, and biology [2]. NPs are particles with dimensions ranging from 1 to 100 nm and are a key topic in nanotechnology. Traditional methods of synthesizing NPs using noble metals like gold, silver, or platinum often involve environmentally unfriendly processes [3]. Therefore, there is a pressing need for the development of non-toxic and environmentally friendly nanoparticle production technologies [4]. In recent years, the safety-by-design principle has driven the development of safe, simple, economical, repeatable, and scalable green synthesis methods for NPs [5].

These methods utilize biological systems, such as plants, algae, yeast, fungi, and bacteria, to synthesize NPs in an environmentally friendly manner [6]. In recent years, the use of different algae species for the biological synthesis of nanoparticles has been trending rapidly. Diverse photosynthetic eukaryotic organisms are independently evolved into different lineages making up the category known as algae. They can produce their food by using light, water, carbon dioxide, or other molecules in the process of photosynthesis [7]. Based on their morphological characteristics, algae are mainly divided into the following two groups: macroalgae and microalgae. They may be found on moist rocks, in freshwater, and in marine environments [8].

There are the following major types of algae in the marine environment: Chlorophyceae (green), Phaeophyceae (brown), Cyanophyceae (blue-green), Rhodophyceae (red), and diatoms [9]. The utilization of algae in nanoparticle synthesis is advantageous for several reasons. Firstly, algae are easy to handle, making the synthesis process more convenient. Secondly, algae have the ability to absorb and accumulate inorganic metallic ions, which is beneficial for NP synthesis and offers a cost-effective alternative compared to traditional methods. Lastly, these biological synthesis methods are naturally eco-friendly, and fast, and promote healthier synthesis practices. In the current study, *Spirulina platensis* is used for the green synthesis of cerium oxide nanoparticles CeO-NPs. *Spirulina platensis* is a type of cyanobacteria that floats freely and has filamentous structures called trichomes. These trichomes are multicellular and have one open end, forming a left-handed helix [10]. *Spirulina plantensis* is known for its high content of vegetable protein, comprising 60-70% of its composition. Recent reports have highlighted the presence of a wide range of minerals and amino acids in the blue-green algae species *Spirulina platensis *[11].

The components are beneficial in reducing and capping nanoparticles. Algal-mediated NPs are a valuable source of biomass for a variety of applications, including those in agriculture, aquaculture, medicine, cosmetics, biotechnology [12], and nanotechnology [13]. Cerium oxide nanoparticles (NPs) have drawn a lot of interest in the field of nanomedicine due to their promising applications in biosensing, drug transport, catalysis, and medicine [14]. These nanoparticles exhibit relative stability, excellent biocompatibility with minimal to no toxicity, cost-effectiveness, and environmental friendliness. Cerium, the main component of CeO_2_, can exist in two different forms, namely Ce_2_O_3_(Ce^3+^) and CeO_2 _(Ce^4+^), depending on the material‘s characteristics [15]. CeO_2_-NPs possess a cubic fluorite structure, with both Ce^3+^ and Ce^4+^ coexisting on their surface. A significant advantage of cerium dioxide is its ability to create oxygen vacancies within its lattice. This property enhances the redox properties of CeO_2_-NPs, making them beneficial for various diseases associated with oxidative stress-related issues [16].

Currently, physical and chemical synthesis are the two main ways used to synthesize CeO_2_ nanoparticles [17]. These techniques, though, use hazardous reducing solvents, which pose a number of risks to the ecology and biodiversity. Additionally, the NPs synthesized by such methods are poisonous and unstable, which reduces their effectiveness [18]. As a result, researchers have recently employed a risk-free, less harmful technique called "green synthesis." This technique makes use of a variety of biological resources, including plants, microorganisms, and other biological products [19].

These biological extracts compose a variety of phytochemicals that have the role of stabilizing the bulk salts and reducing them into the corresponding nanoparticle synthesis [19]. Numerous uses of green synthesized CeO-NPs, including antibacterial, anticancer, anti-larvicidal, photocatalytic, and antioxidant therapy [20], have been described to date [21]. The current study focuses on producing cerium oxide nanoparticles through Spirulina. Due to the green synthesis method, they contain phytochemicals that are biocompatible, nontoxic, and environmentally benign. They are further characterized using UV spectrometry, scanning electron microscopy (SEM), energy-dispersive X-ray (EDX), Fourier transform infrared spectroscopy (FT-IR), and X-ray diffraction analysis (XRD). This study examines the antimicrobial and anti-inflammatory properties of cerium oxide nanoparticles.

## Materials and methods

Materials and reagents

Metal oxide was purchased from Sisco Research Laboratories (Mumbai, India), cerium nitrate hexahydrate was purchased from Loba Chemie Pvt Ltd (Mumbai, India), and nutrient agar and Mueller-Hinton (MH) agar were purchased from Hi-Media (Mumbai, India). The four bacterial cultures were obtained from the Department of Microbiology at Saveetha Medical College and Hospitals (Chennai, India).

Synthesis of nanoparticles

*Spirulina* *platensis* was collected from Kovalam, Tamil Nadu, it was taxonomically identified by Professor N Siva, Department of Botany, Raja Doraisingam Government Arts College Sivagangai, Tamil Nadu. The Spirulina was air-dried after being three times rinsed in distilled water and was allowed to air dry. A mechanical grinder (Nanchang Kay Xin Yue Technologies Co., Jiangxi, China) was used to powder the dry sample. A combination of 10 g of Spirulina powder and 200 mL of distilled water was made and then autoclaved. After that, the extract was kept in storage at 4°C, and the solution was then filtered using Whatman filter paper grade no. 1.

An aqueous extract of 150 mL was mixed drop by drop with 25 mL of 25 mM cerium nitrate hexahydrate (Ce {NO_3_}₂·6 H₂O) using the titration method, and the mixture was shaken throughout the night for incubation. After an entire night of incubation, the color shift was noticed from dark black to brown. After that the mixture was centrifuged at 4500 RPM for 30 min and the pellet was collected, and stored in an airtight container at ambient temperature for further characterization studies.

Characterization techniques

UV-visible spectroscopy was used to examine the optical characteristics of the nanoparticle synthesis using a LMSPUV1900S - UV-Vis. Double Beam Spectrophotometer (Chennai, India: LABMAN) and 1 cm quartz cuvettes in the 200-1000 nm range. FT-IR examination in the 500-3500/cm range was performed using Bruker Alpha II (Ettlingen, Germany) to investigate the role of different biomolecules that act as capping, reducing, and stabilizing agents in the synthesis of CeO-NPs. XRD is commonly applied for analyzing molecular and crystal structures, a qualitative identification and resolution of active compounds and different molecules, and measurement of crystallinity, isomorphous substitution, and particle size. The phytochemical characteristics of the crystal lattice are represented by the abundance of diffraction peak values that are produced when X-ray radiation is reflected on any particles. XRD is used to investigate the structural properties of different materials, inorganic catalysts, superconductors, biomolecules, glasses, and polymers. Employing scanning electron microscopy (SEM) techniques on a JSM-7001F (Tokyo, Japan: JEOL Ltd) working at an accelerating voltage of 20 kV, the surface morphology of the synthesized CeO-NPs was investigated. The elements of the doped nanoparticles were examined using an Energy dispersive X-ray (EDX) spectrometer (X-Plor-30/C-Swift; Abingdon, UK: Oxford Instruments) heated at 10°C/min in a nitrogen environment.

Antibacterial activity

The biosynthesized CeO-NPs using Spirulina were tested against antibacterial activity using the agar well diffusion method by utilizing Mueller Hinton agar (MHA) plates. The tested organisms (*Escherichia coli*, methicillin-resistant *Staphylococcus aureus* (MRSA), and *Pseudomonas aeruginosa*) were uniformly swabbed on MHA plates. The biosynthesized CeO-NPs (size 75-125 nm) samples were prepared in different concentrations (20, 40, 60, and 80 µg/mL) and poured into 6 mm wells created in the inoculated media using sterile tips. Streptomycin (30 µg/mL) was used as a positive control and dimethyl sulfoxide (DMSO) was used as a negative control. After allowing the extracts to diffuse for approximately 30 minutes at room temperature, the plates were incubated at 37°C for 18-24 h. Following incubation, the plates were examined for the presence of clear zones around the wells, indicating the antibacterial activity of the tested compounds. The diameter of these zones of inhibition (ZOI) was measured in millimeters. Using the zone of inhibition results, minimum inhibitory concentration was determined by antibacterial susceptibility testing according to Clinical and Laboratory Standards Institute (CLSI) guidelines.

Anti-inflammatory activity

The albumin denaturation method was slightly modified to study the anti-inflammatory results. The reaction mixture includes (20, 40, 60, 80, 100 µg/mL) of Bovine serum albumin and (80, 60, 40, 20 µg/mL) of CeO-NPs in different concentrations. The concentrations at the terminal points rise to 20, 40, 60, 80, and 100 µg/mL. Diclofenac sodium was used as standard and DMSO was used as a negative control drug in a comparable volume. After incubating for 15 minutes at 37°C, the reaction mixture was maintained for 20 min at 55°C. The absorbance was then measured at 660 nm. We used the following formula to calculate inhibition. Percentage inhibition = (control of absorbance - sample of absorbance) × 100/control of absorbance).

Statistical analysis

The data were entered in Microsoft Excel 2016 (Redmond, WA: Microsoft) and subjected to statistical analysis, reported as mean±standard deviations for triplicate experiments. Statistical significance was set at p<0.05 and analyzed by one-way ANOVA.

## Results

Synthesis of CeO-NPs from Spirulina aqueous extract

**Figure 1:**
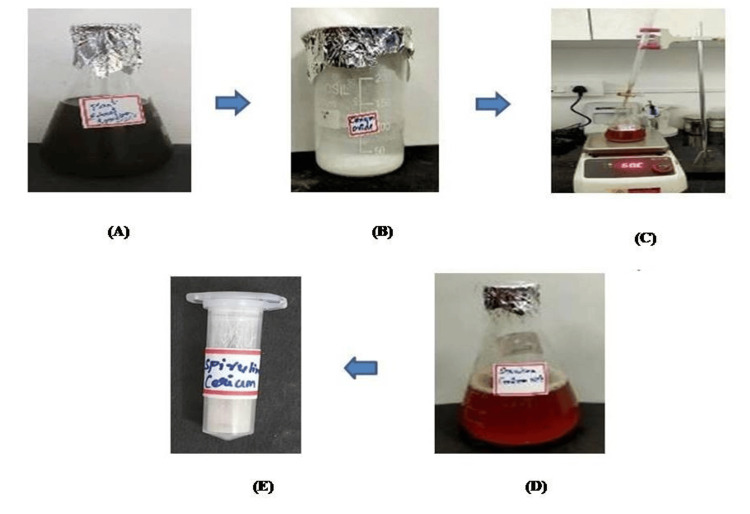
Synthesis of Spirulina-mediated CeO-NPs. Synthesis of CeO-NPs from Spirulina (A) 150 mL of aqueous extract of Spirulina, (B) 25 mM of cerium nitrate hexahydrate, (C) titration method, (D) formation of CeO-NPs after 24 h of incubation, and (E) the powder form of CeO-NPS. CeO-NPs: cerium oxide nanoparticles

The results demonstrate that the aqueous extract of Spirulina serves as an effective reducing and capping agent, due to its bioactive compounds. The observed color change from dark brown to reddish brown following a 24-h incubation period indicates a successful reduction of cerium ions observed in Figures 1A-1D. This color transformation is indicative of the underlying chemical reaction, wherein cerium ions are reduced, likely leading to the formation of cerium nanoparticles. The color alteration validates the Spirulina extract's reducing activity and underscores its potential applications in the synthesis of nanomaterials and related fields.

Characterization of CeO-NPs UV-Vis spectroscopy

**Figure 2:**
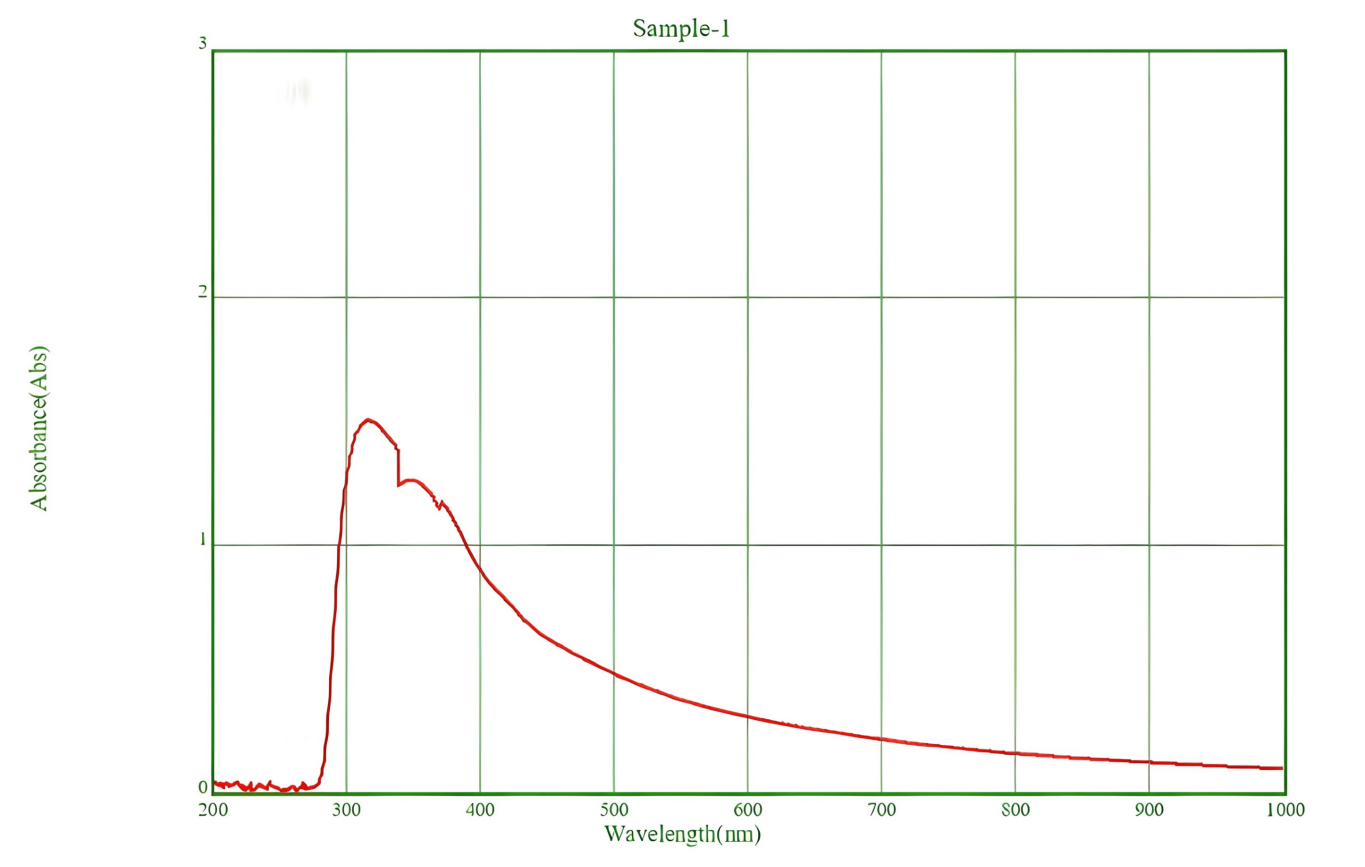
UV-vis absorption spectrum of the CeO-NPs suspension. UV-vis: UV-visible; CeO-NPs: cerium oxide nanoparticles

The characterization of CeO-NPs biosynthesized using Spirulina was conducted through UV-visible spectroscopy, revealing a significant absorption peak at approximately 320 nm (Figure 2). This peak is indicative of light absorption by the CeO-NPs.

Fourier transform infrared spectroscopy (FT-IR)

**Figure 3:**
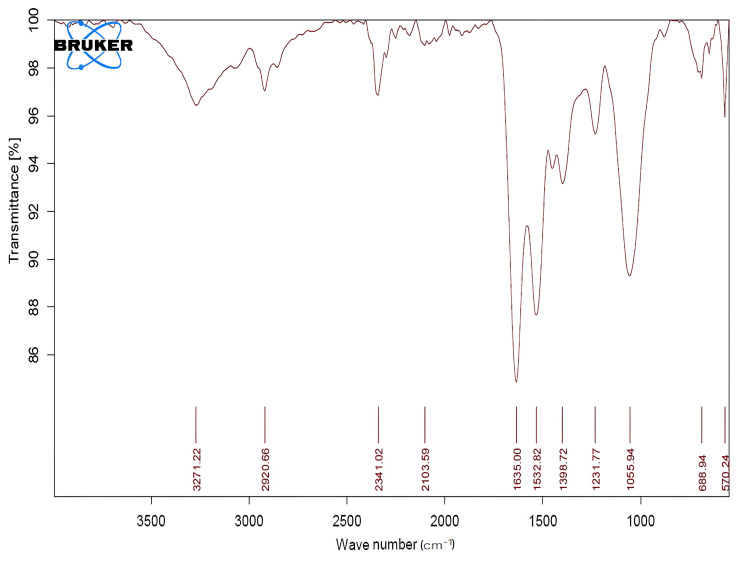
Fourier transformed infrared spectra of the synthesized CeO-NPs. CeO-NPs: cerium oxide nanoparticles

The synthesized CeO-NPs functional groups were identified using FT-IR. Four functional groups were determined based on the absorption peaks observed in the spectra. These peaks were absorbed in 1055.94/cm corresponding to the C-O stretching group, 1532.82/cm to the N-O stretching group, and 1635.00/cm to the C=C stretching group (Figure 3, Table 1).

**Table 1:** FT-IR analysis of CeO-NPs. FT-IR: Fourier transform infrared spectroscopy; CeO-NPs: cerium oxide nanoparticles

Frequency per cm	Absorption per cm	Group	Compound
2000-1000	1635.00, 1532.82, 1055.94	C=C stretching group, N-O stretching group, C-O stretching group	Alkenyl group
500-1000	570.24, 688.94	C-I stretch, C-Br stretch	Aliphatic iodo compounds, aliphatic bromo compounds

X-ray diffraction analysis (XRD)

**Figure 4:**
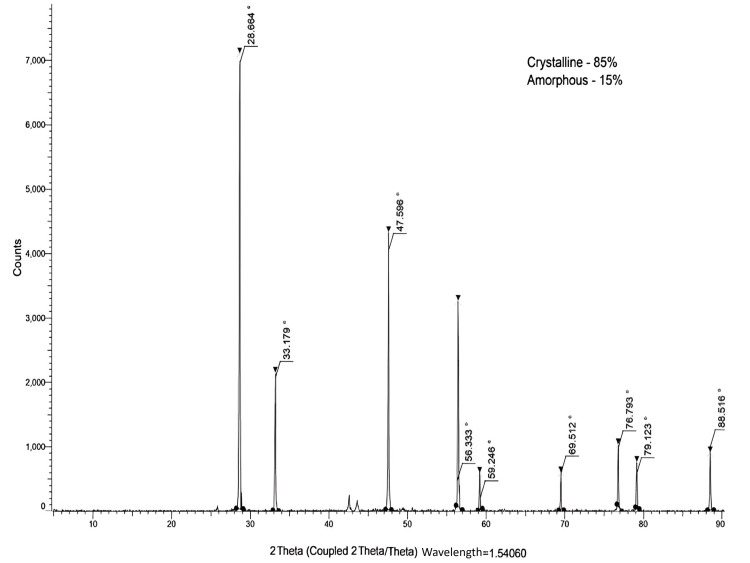
X-ray powder diffraction spectra Spirulina mediated CeO-NPs. CeO-NPs: cerium oxide nanoparticles

The XRD pattern of the biosynthesized CeO-NPs indicates a high degree of crystallinity, quantified at 85%, alongside a relatively low proportion of amorphous characteristics at 15% (Figure 4). The presence of distinct diffraction peaks at 2θ positions of 28.6°, 33.17°, 47.5°, 56.3°, 59.2°, 69.5°, 76.6°, 88.5° further supports the conclusion that the synthesized CeO-NPs possess a stable crystalline structure. These peaks correspond to the characteristic reflections of cerium oxide, confirming the successful synthesis of crystalline CeO-NPs. The high crystallinity suggests that the nanoparticles are well-ordered, which is advantageous for their potential applications in various fields. Thus, the synthesized CeO-NPs have a very stable crystalline structure.

Scanning electron microscopy (SEM)

**Figure 5:**
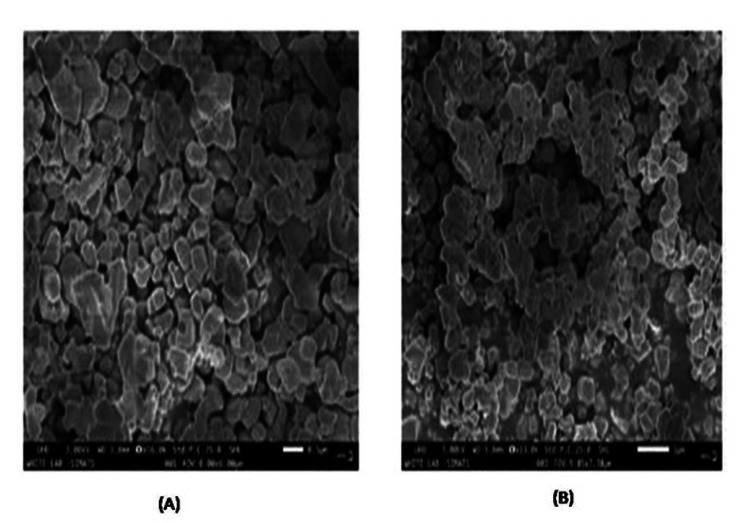
SEM images of prepared CeO-NPs with different diameter distribution - (A) 0.5 µm scale and (B) 1 µm scale. SEM: scanning electron microscopy; CeO-NPs: cerium oxide nanoparticles

SEM was used to examine the surface morphology and chemical composition of CeO-NPs synthesized at various magnifications. The biosynthesized CeO-NPs exhibited square shapes with an agglomerated structure, and their average size ranged from 75-125 nm (Figures 5A, 5B).

Energy dispersive X-ray (EDX) analysis

**Figure 6:**
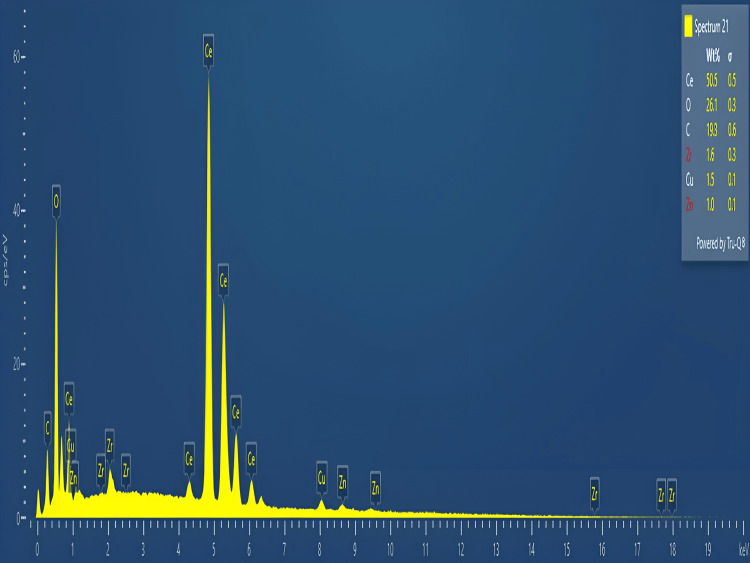
EDX images of synthesized CeO-NPs. EDX: energy dispersive X-ray; CeO-NPs: cerium oxide nanoparticles

The elemental constituents of CeO-NPs were determined by using EDX analysis. The EDX results showed signals for Ce (50.5%), O (26.1%), and C (19.3%) (Figure 6).

Antibacterial activity

**Figure 7:**
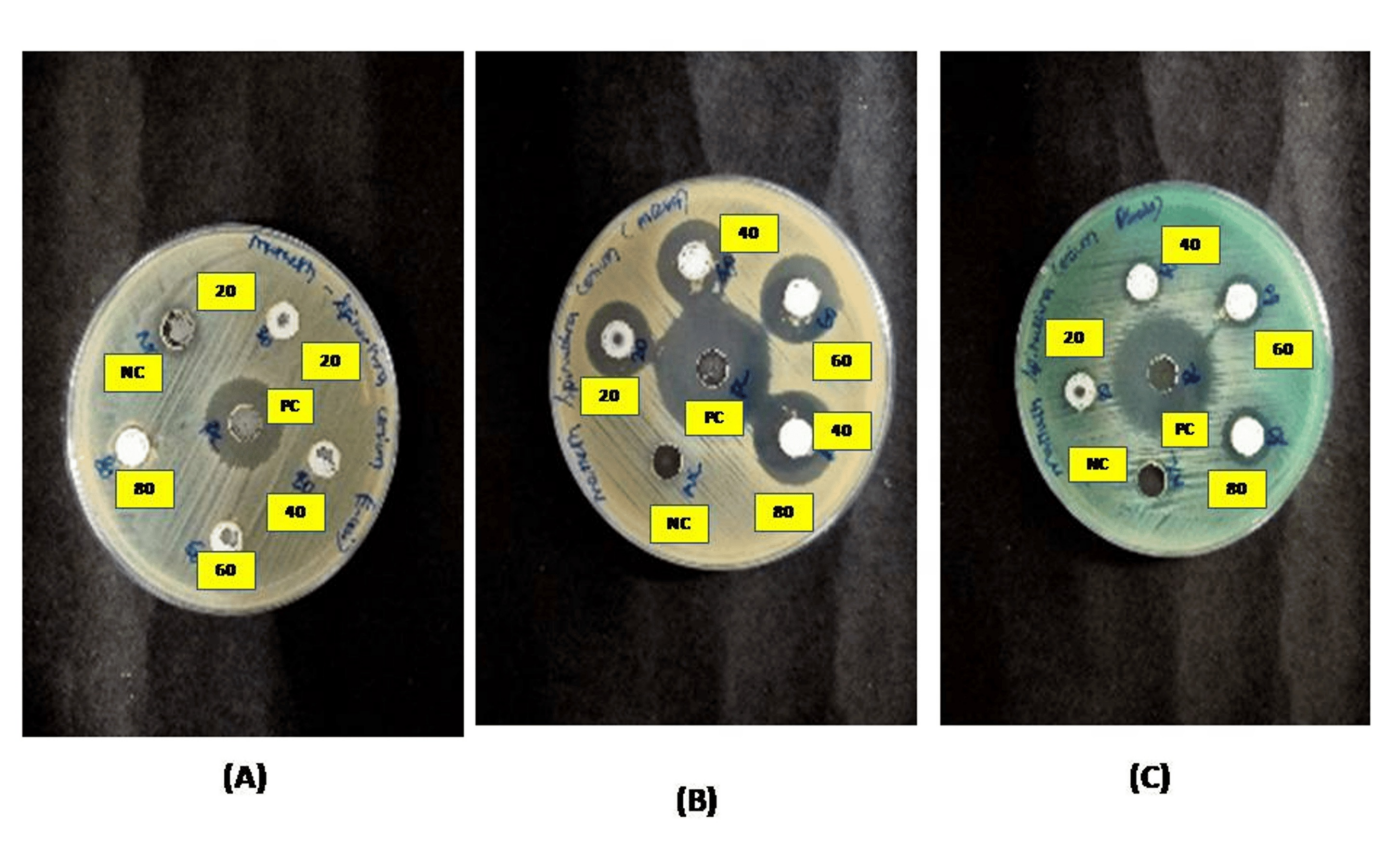
Antibacterial activity of CeO-NPs from Spirulina - (A) E. coli, (B) MRSA, (C) Pseudomonas aeruginosa. CeO-NPs: cerium oxide nanoparticles; MRSA: methicillin-resistant *Staphylococcus aureus*

The antibacterial activity of synthesized CeO-NPs evaluated against different strains depicted in Figures 7A-7C. The zone of inhibition of CeO-NPs was summarized in various concentrations (20, 40, 60, 80 µg/mL). The ZOI values recorded for CeO-NPs were *Pseudomonas aeruginosa* (11, 12, 13, and 14 mm), MRSA (16, 17, 19, and 20 mm), and there was no zone of inhibition against *Escherichia coli.*

Minimum inhibitory concentration

The minimum inhibitory concentration (MIC) results indicate that CeO-NPs exhibit varying levels of antibacterial effectiveness against the tested bacterial strains. Specifically, the highest concentration required to inhibit the growth of *Escherichia coli *(500 µg/mL), suggesting a lower sensitivity to CeO-NPs. In contrast, *Pseudomonas aeruginosa* shows a moderate sensitivity, with an MIC of 125 µg/mL. Notably, MRSA demonstrates the highest sensitivity to CeO-NPs. In contrast, *Pseudomonas aeruginosa* shows a moderate sensitivity, with an MIC of 125 µg/mL. Notably, MRSA demonstrates the highest sensitivity to CeO-NPs, with a MIC of just 31.25 µg/mL. These results suggest that CeO-NPs are particularly effective against MRSA compared to the other strains tested (Table 2).

**Table 2:** Minimum inhibitory concentration of CeO-NPs. CeO-NPs: cerium oxide nanoparticles

S. no.	Organism name	Minimum inhibitory concentration (MIC) (µg/mL)
1	E. coli	500
2	Pseudomonas aeruginosa	125
3	MRSA	31.25

Anti-inflammatory activity

**Figure 8:**
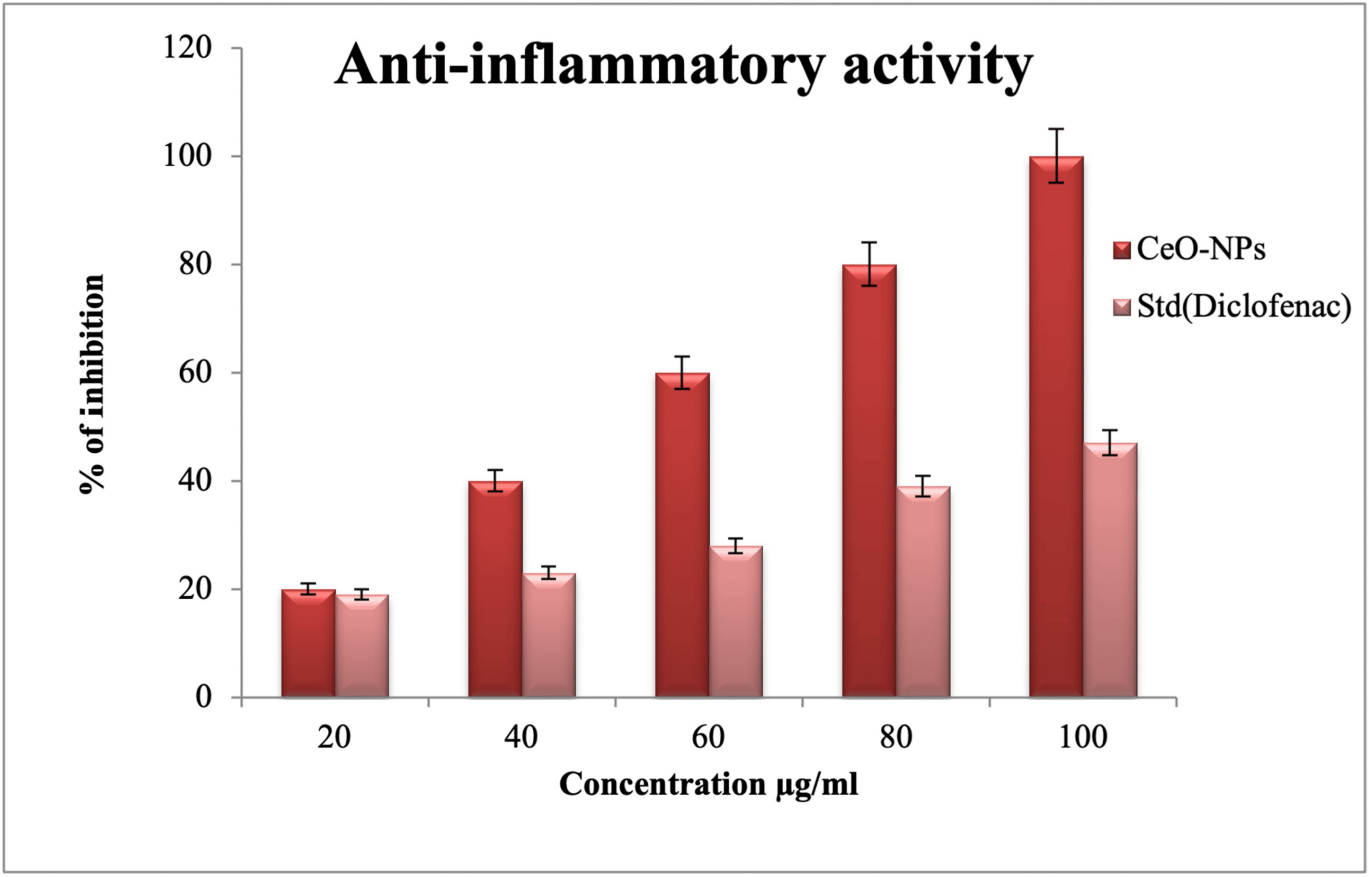
Anti-inflammatory activity of CeO-NPs. CeO-NPs: cerium oxide nanoparticles; Std.: standard

The anti-inflammatory activity of the synthesized CeO-NPs was found to vary in a dose-dependent manner. Among the concentrations tested, the highest concentration of 100 µg/mL exhibited the highest level of anti-inflammatory activity (47%). On the other hand, the lowest concentration of 20 µg/mL displayed a lower level of anti-inflammatory activity (19%). At a concentration of 60 µg/mL, a moderate level of activity (28%) was observed. This observation is based on the concept that increasing the concentration of CeO-NPs or the standard diclofenac sodium leads to increased anti-inflammatory activity. Conversely, decreasing the concentration results in a decrease in activity (Figure 8).

## Discussion

*Spirulina platensis*, also known as Arthospira, is a type of blue-green algae that contains various phytochemical compounds, including amino acids, alkaloids, phenolic compounds, flavonoids, and other metabolites, which are present in the aqueous extract of Spirulina. The bioactive compounds present in the Spirulina extract act as reducing and capping agents [11]. After the completion of the 24-h incubation period, the color change was observed from dark brown to reddish brown (Figures 1A-1D). The color change was obtained because of the reduction of cerium ions. Comparatively, the green synthesis of *Colocasia esculenta* leaf extract showed that gradual reduction of Ce^4+ ^to Ce⁰ [22]. As per the earlier research, this justified the synthesis of CeO-NPs.

The biosynthesis of CeO-NPs using Spirulina was characterized by a UV-visible spectrum, which showed a peak at approximately 320 nm. The findings align with previous research, which identified a similar absorption peak at 324 nm for CeO_2_-NPs, attributed to the photoexcitation of electrons from the valence band to the conduction band [23]. Furthermore, UV results of *Colocasia esculenta*​​​​​​, indicated an absorbance peak at 213 nm suggesting the formation of CeO_2_-NPs [22]. The CeO_2_-NPs were synthesized using *Gloriosa superba *lily leaf extract, which exhibited an absorption peak at 297 nm. Collectively, these findings confirm that the synthesized nanoparticles are CeO-NPs [24].

The functional groups present in the synthesized CeO-NPs were characterized using FT-IR revealing four distinct absorption peaks. The peaks were observed at 1055.94/cm(C-O stretching group), 1532.82/cm (N-O stretching group), and 1635.00/cm​​​​​​​ (C=C stretching group). These findings suggest the presence of various organic compounds associated with the Spirulina extract that may play a role in the stabilization and capping of the nanoparticles. In comparison to a similar study involving *Moringa oleifera* peel extract, which exhibited the absorption bands at 3279/cm, 2919/cm, 1593/cm, and 1035/cm, the differences in peak positions indicate variations in the functional groups present in the two extracts. Notably, the vibrations observed at 507/cm and 1040/cm in the *Moringa oleifera* study were attributed to Ce-O bonds, further supporting the formation of cerium oxide nanoparticles [25]. Overall, the FT-IR analysis confirms the successful synthesis of CeO-NPs and highlights the presence of specific functional groups that may contribute to the nanoparticles' stability and functionality.

The XRD pattern of the biosynthesized CeO-NPs showed a higher degree of crystallinity (85%) and a lower proportion of amorphous characteristics (15%), with diffraction peaks at 2θ positions of 28.6°, 33.17°, 47.5°, 56.3°, 59.2°, 69.5°, 76.6°, 88.5°. A similar study also reported the presence of specific peaks, such as 1,1,1; 2,0,0; 2,2,0; 3,1,1; 2,2,2; 4,0,0; 3,3,1; and 4,2,0, in the XRD pattern, which corresponds to the crystal planes of CeO_2_-NPs [24]. These findings suggest that the synthesized CeO-NPs are pure and exhibit a crystalline nature. Similarly, strong and prominent diffraction peaks at 2θ locations of 28.43°, 33.62°, 48.38°, 57.7°, 59.03°, 69.37°, and 76.6° [26]. The XRD results revealed that the synthesized CeO-NPs possess a highly stable crystalline structure.

The results from the SEM analysis indicate that the biosynthesized CeO-NPs exhibit distinct morphological characteristics. The observed square shapes and agglomerated structure, with an average size ranging from 75-125 nm, suggest that the synthesis conditions may favor the formation of these specific geometries. This contrasts with findings from the previous study, where CeO_2_-NPs synthesized using *Abelmoschus esculentus* extract displayed a more uniform distribution and smaller average size of 35-40 nm [27]. The transformation in particle morphology with increased temperature is particularly significant. As the temperature rises, the square-shaped particles with significant agglomeration transition to a more spherical shape with reduced agglomeration [28]. This change may indicate that higher temperatures facilitate better dispersion and a more uniform growth of the nanoparticles, leading to a decrease in agglomeration and a shift in shape. Overall, these findings highlight the influence of synthesis conditions, such as temperature, on the morphology and size of cerium oxide nanoparticles. The EDX analysis of CeO-NPs revealed the following elemental composition: cerium (Ce) constituted 50.5% of the sample, indicating it is the primary component, and oxygen (O) accounted for 26.1%, which is consistent with the formation of cerium oxide nanoparticles. Additionally, carbon (C) was detected at 19.3%, which may suggest the presence of organic residues or contaminants in the sample. These results provide insight into the elemental makeup of the nanoparticles, confirming the successful synthesis of CeO-NPs with the expected constituents.

The antibacterial activity of CeO-NPs showed significantly higher antibacterial activity against MRSA and *Pseudomonas aeruginosa* and lower activity against *E. coli*. The antibacterial properties of CeO-NPs are primarily influenced by factors, such as their size, area of surface, and topology [15]. Furthermore, the formation of reactive oxygen species (ROS) within the bacterial cells may be facilitated by the electrostatic interaction between positively charged nanoparticles and negatively charged bacterial cells. In our current study, the Ce^+^ nano ion can enter the bacterial cell wall, further, it leads to the result of DNA damage, inhibition of bacterial growth, and eventually cell death [29].

CeO nanoparticles (CeO-NPs) synthesized by us demonstrated potential activity in a concentration-dependent manner, achieving a maximum inhibition of 47% at a concentration of 100 µg/mL. Furthermore, CeO and Mg-doped nanoparticles derived from *Hibiscus sabdariffa *exhibited significantly high anti-inflammatory activity at a maximum concentration of 500 µg/mL [30]. In contrast, our findings indicated substantial anti-inflammatory activity at a lower concentration of 100 µg/mL [28]. This implies that our synthesized CeO-NPs are more effective in terms of anti-inflammatory activity compared to another study, which reported a maximum inhibition of 52% at a higher concentration of 500 µg/mL.

Limitations

This study offers significant insights into the antibacterial and anti-inflammatory properties of Cerium oxide nanoparticles synthesized from Spirulina. However, it is essential to assess the durability and toxicity of these nanoparticles to explore their potential therapeutic applications. The focus on short-term observations in the research hinders a thorough understanding of the nanoparticles' stability and long-term effects.

## Conclusions

The synthesis of CeO-NPs using Spirulina as a mediator has shown promising results in terms of their antibacterial and anti-inflammatory efficacy. The Spirulina-mediated synthesis method has facilitated the synthesis of CeO-NPs with desirable properties, such as size, surface morphology, and elemental composition. Additionally, the CeO-NPs have exhibited antibacterial and anti-inflammatory effects, which could potentially contribute to their therapeutic applications. Further research and studies are warranted to explore the full potential of Spirulina-mediated CeO-NPs in antibacterial and anti-inflammatory treatments.

## References

[1] Harikrishnan A, Ramalingam B, Nadeem A, Ramachandran B, Veena VK, Muthupandian S (2024). Eco-friendly synthesis of zinc oxide nanoparticles (ZnOnps) from Piper betel leaf extract: spectral characterization and its application on plant growth parameters in maize, fenugreek and red gram. Mater Tech.

[2] Marthandan PA, Sravanthy PG, Snega R, Carmelin D, Surya M, Venugopal DC, Saravanan M (2024). Melothria maderaspatana mediated one-pot synthesis of cerium-doped Silymarin nanoparticles and their antibacterial and anticancer studies. Mater Tech.

[3] Sumaira Sumaira, Khan T, Abbasi BH (2017). Melatonin-enhanced biosynthesis of antimicrobial AgNPs by improving the phytochemical reducing potential of callus culture of Ocimum basilicum L. var. thyrsiflora. RSC Adv.

[4] Devi NS, Ganapathy DM, Rajeshkumar S, Maiti S (2022). Characterization and antimicrobial activity of cerium oxide nanoparticles synthesized using neem and ginger. J Adv Pharm Technol Res.

[5] Shah M, Nawaz S, Jan H (2020). Synthesis of bio-mediated silver nanoparticles from Silybum marianum and their biological and clinical activities. Mater Sci Eng C Mater Biol Appl.

[6] Anandalakshmi K (2021). Green synthesis of silver nanoparticles using plant extracts - a review. Plant Archiv.

[7] Tripathi DK, Tripathi A, Shweta Shweta (2017). Uptake, accumulation, and toxicity of silver nanoparticle in autotrophic plants, and heterotrophic microbes: a concentric review. Front Microbiol.

[8] Sharma D, Kanchi S, Bisetty K (2019). Biogenic synthesis of nanoparticles: a review. Arabian J of Chem.

[9] Wang HD, Li XC, Lee DJ, Chang JS (2017). Potential biomedical applications of marine algae. Bioresour Technol.

[10] Doshi H, Ray A, Kothari IL (2007). Bioremediation potential of live and dead Spirulina: spectroscopic, kinetics and SEM studies. Biotechnol Bioeng.

[11] Vasanth V, Murugesh KA, Tilak M, Aruna R, Raj PM, Arasakumar E (2023). Green synthesis of Spirulina mediated titanium dioxide nanoparticles and their characterization. J Surv Fish Sci.

[12] Gungor AA, Nadaroglu H, Babagil A, Babagil A, Onem H (2018). Green synthesis of nanoceria (CeO2) and evaluation of enzyme-like characteristics. J Adv Mater Proc.

[13] Ha HA, Al-Ansari MM, Al-Dahmash ND, Krishnan R, Shanmuganathan R (2023). In vitro analyses of cerium oxide nanoparticles in degrading anthracene/fluorene and revealing the antibiofilm activity against bacteria and fungi. Chemosphere.

[14] Nadeem M, Khan R, Afridi K (2020). Green synthesis of cerium oxide nanoparticles (CeO2 NPs) and their antimicrobial applications: a review. Int J Nanomedicine.

[15] Singh S, Jain RK (2024). The synthesis, characterization, and assessment of antibacterial properties of an orthodontic adhesive containing cerium-substituted hydroxyapatite nanoparticles: an In vitro study. Cureus.

[16] Das S, Dowding JM, Klump KE, McGinnis JF, Self W, Seal S (2013). Cerium oxide nanoparticles: applications and prospects in nanomedicine. Nanomedicine (Lond).

[17] Rajeshkumar S, Naik P (2018). Synthesis and biomedical applications of Cerium oxide nanoparticles - a review. Biotechnol Rep (Amst).

[18] Choudary MR, Surya M, Saravanan M (2024). Green synthesis of cerium oxide nanoparticles using Tribulus terrestris: characterization and evaluation of antioxidant, anti-inflammatory and antibacterial efficacy against wound isolates. Biomed Phys Eng Express.

[19] Arunachalam T, Karpagasundaram M, Rajarathinam N (2017). Ultrasound assisted green synthesis of cerium oxide nanoparticles using Prosopis juliflora leaf extract and their structural, optical and antibacterial properties. Mater Sci Pol.

[20] Nadaroglu H, Onem H, Alayli Gungor A (2017). Green synthesis of Ce(2)O(3) NPs and determination of its antioxidant activity. IET Nanobiotechnol.

[21] Kumar KM, Mahendhiran M, Diaz MC (2018). Green synthesis of Ce3+ rich CeO2 nanoparticles and its antimicrobial studies. Mater Lett.

[22] Miri A, Darroudi M, Sarani M (2020). Biosynthesis of cerium oxide nanoparticles and its cytotoxicity survey against colon cancer cell line. Appl Organomet Chem.

[23] Ahmad NM, Hasan NA (2023). Synthesis of green cerium oxide nanoparticles using plant waste from Colocasia esculenta for seed germination of mung bean (Vigna radiata). J Nanotechnol.

[24] Wang W, Zhang B, Jiang S, Bai H, Zhang S (2019). Use of CeO2 nanoparticles to enhance UV-shielding of transparent regenerated cellulose films. Polymers (Basel).

[25] Arumugam A, Karthikeyan C, Haja Hameed AS, Gopinath K, Gowri S, Karthika V (2015). Synthesis of cerium oxide nanoparticles using Gloriosa superba L. leaf extract and their structural, optical and antibacterial properties. Mater Sci Eng C Mater Biol Appl.

[26] Surendra TV, Roopan SM (2016). Photocatalytic and antibacterial properties of phytosynthesized CeO2 NPs using Moringa oleifera peel extract. J Photochem Photobiol B.

[27] Ahmed HE, Iqbal Y, Aziz MH (2021). Green synthesis of CeO(2) nanoparticles from the Abelmoschus esculentus extract: evaluation of antioxidant, anticancer, antibacterial, and wound-healing activities. Molecules.

[28] Mohanraj VJ, Chen Y (2006). Nanoparticles - a review. Trop J Pharm Res.

[29] Zhang M, Zhang C, Zhai X, Luo F, Du Y, Yan C (2019). Antibacterial mechanism and activity of cerium oxide nanoparticles. Sci China Mater.

[30] Alghamdi AA (2023). Biogenic Mg doped CeO2 nanoparticles via Hibiscus sabdariffa and its potential biological applications. J Umm Al-Qura Univ Appll Sci.

